# The Pheromone of the Cave Cricket, *Hadenoecus cumberlandicus*, Causes Cricket Aggregation but Does Not Attract the Co-Distributed Predatory Spider, *Meta ovalis*

**DOI:** 10.1673/031.010.4701

**Published:** 2010-05-13

**Authors:** Jay A. Yoder, Brady S. Christensen, Travis J. Croxall, Justin L. Tank, Horton H. Hobbs

**Affiliations:** Department of Biology, Wittenberg University, Springfield, OH 45501 USA

**Keywords:** antipredator behavior, arresting, clustering, guano, shared habitat, uric acid

## Abstract

Food input by the cave cricket, *Hadenoecus cumberlandicus* Hubble & Norton (Orthoptera: Rhaphidophoridae), is vital to the cave community, making this cricket a true keystone species. Bioassays conducted on cave walls and in the laboratory show that clustering in *H. cumberlandicus* is guided by a pheromone, presumably excreta. This aggregation pheromone was demonstrated by using filter paper discs that had previous adult *H. cumberlandicus* exposure, resulting in > 70% response by either nymphs or adults, prompting attraction (thus, active component is a volatile), followed by reduced mobility (arrestment) on treated surfaces. Adults were similarly responsive to pheromone from nymphs, agreeing with mixed stage composition of clusters in the cave. Effects of [0.001M – 0.1M] uric acid (insect excreta's principle component) on *H. cumberlandicus* behavior were inconsistent. This pheromone is not a host cue (kairomone) and is not used as a repellent (allomone) as noted through lack of responses to natural *H. cumberlandicus* pheromone and uric acid concentrations by a co-occurring predatory cave orb weaver spider, *Meta ovalis* Gertsch (Araneae: Tetragnathidae). This pheromone is not serving as a sex pheromone because nymphs were affected by it and because this population of *H. cumberlandicus* is parthenogenic. The conclusion of this study is that the biological value of the aggregation pheromone is to concentrate *H. cumberlandicus* in sheltered sites in the cave conducive for minimizing water stress. Rather than signaling *H. cumberlandicus* presence and quality, the reduced mobility expressed as a result of contacting this pheromone conceivably may act as a defense tactic (antipredator behavior) against *M. ovalis*, which shares this favored habitat site.

## Introduction

In the twilight zone of caves, within recessed areas of walls and ceiling depressions the cave orb weaver spider, *Meta ovalis* Gertsch (Araneae: Tetragnathidae), is frequently observed coinciding with the presence of large roosting aggregations of the cave cricket, *Hadenoecus cumberlandicus* Hubble & Norton (Orthoptera: Rhaphidophoridae). In addition, *H. cumberlandicus* and *M. ovalis* can be found out in the open, at wall/ceiling junctures. This cave cricket-spider cooccurrence is so regular, in fact, that the presence of one typically is used to indicate the presence of the other in survey work; the large, easily noticed *M. ovalis*, in particular, is especially useful in this regard as a landmark for pointing out large aggregations of *H. cumberlandicus* (mixed stages) that would otherwise be concealed in expanded ceiling fractures or joints overhead. This codistribution between *H. cumberlandicus* and *M. ovalis* is not only observed in many of the caves in northeastern Kentucky (Carter County), USA, but also throughout the majority of cave systems in North America where cave crickets (*Hadenoecus* spp.) and *M. ovalis* are found (Hill 2003; Lavoie et al. 2007; HH Hobbs III, unpublished data). The large orb weaver spider, *M. ovalis*, although not present in all of these caves, is recognized as an *H. cumberlandicus* predator (Lavoie et al. 2007). On the other hand, the cave cricket, *Hadenoecus* spp., is more ubiquitous and is a dominant member of the cave community as a keystone species, providing a predictable source of guano that supplies nutrients (both directly and indirectly) to an array of springtails, beetles, flies, and millipedes that thrive below the *Hadenoecus* roost on the cave floor (Poulson et al. 1995). Thus, *M. ovalis* are commonly found at sites where *H. cumberlandicus* aggregate.

The size of the aggregation usually consists of 5–10 individuals per cluster but may reach densities greater than 200 individuals (Hobbs 1992; Hill 2003). Relative humidity is high, approximately 97% RH, temperature is moderate, averaging 15° C, and dry air currents are reduced in kettles (or bells) in the upper walls and ceiling of the cave where these aggregations are formed (Hobbs 1992). Selection of these stable, humid, unventilated sites by *H. cumberlandicus* is highly favorable for maintaining water balance (Studier et al. 1987; Studier and Lavoie 1990) and also for protecting against predation by certain ground-dwelling beetles by keeping *H. cumberlandicus* high above the ground (Peck 1976). The formation of the aggregation itself has the benefit of enhancing water conservation behaviorally via a group effect that further protects the *H. cumberlandicus* from desiccation (Yoder et al. 2002). Lavoie et al. (2007) noted that *H. cumberlandicus* repeatedly use the same sites within the cave for aggregation, presumably one generation after the next. Poulson et al. (1995) and Hill (2003) reported that *H. cumberlandicus* return from the outside after nightly foraging bouts to previously occupied sites within the cave where they, in turn, defecate copiously. As such, these highly specific sites used by *H. cumberlandicus* within the cave are localized regions of heavy guano accumulation.

This study tests the hypothesis that *H. cumberlandicus* is responding to an aggregation pheromone, because of the apparent derivation of this pheromone from feces (i.e. guano), supported by previous work on crickets (Nagel and Cade 1983; McFarlane et al. 1983) and locusts (Byers 1991), site persistence, and resultant expression of aggregation behavior. This study also seeks to examine whether this *H. cumberlandicus* aggregation pheromone, if present, is attractive for *M. ovalis*, which may explain the co-occurrence of this predator in proximity to the *H. cumberlandicus* roost by acting as a host cue (kairomone). It is not uncommon for a predator to use excretory products of prey as a potential signal of healthy prey and their abundance (Weiss 2006) or for predator excreta to be associated with reduced movement by prey to go undetected as a novel form of antipredator behavior (Persons and Rypstra 2001; Kortet and Hedrick 2004; Wilder and Rypstra 2004). As such, uric acid (varying concentrations) is evaluated as one of possible active components mediating the aggregation response in *H. cumberlandicus* because of its high occurrence as a component of excreta in most insects (Nation 2001). Additionally, a combination of laboratory and field experiments were used because of the sensitivity of trogloxenes, especially when taken out of the cave environment, so that it can be determined to what extent laboratory observations reflect how *H. cumberlandicus* may behave in the deep cave environment.

## Materials and Methods

### Crickets, spiders, and test conditions

The study site was the dry upper level of Laurel Cave (N 38° 22′ 30.8″ W 83° 06′ 55.4″) in Carter Cave State Resort Park (Carter County, Kentucky, USA) from August to October, 2003–2007. The study site was in a dry, phreatic, 280 m long passage that was situated above a lower active vadose stream level. Laurel Cave is a multi-entrance cave system developed in Mississippian limestone (total cave length 1091 m). This area is a horizontal, tubular paleo-trunk conduit ranging in height from 1–4 m that intersects the lower stream passage approximately 4.5 m above the stream and about 200 m northwest of a 1 m high × 4 m wide entrance (Pfeffer et al. 1981). There are no active streams, only epikarstic drip input in the study area that has an extensive twilight zone (30+ m), a dark zone, and is easily accessible by *H. cumberlandicus* and *M. ovalis* through cave entrances or crevices leading to the surface. During winter, cold, dry air blows into this level via the entrance, causing *H. cumberlandicus* to move further into the cave; whereas in summer, air blows out of the entrance and *H. cumberlandicus* are not restricted to the deeper confines of the upper level. The area of the cave that was used for these experiments involved a total of five separate study sites within an expanded area of the twilight zone approximately 20 m from the entrance where *H. cumberlandicus* have been shown to be abundant (Hill 2003).

A parthenogenic population of *H. cumberlandicus* exists in Laurel Cave (Hubbell and Norton 1978). Adult *H. cumberlandicus* are distinguished by a large, darkly sclerotized ovipositor and are of larger size than nymphs. Male and female *M. ovalis* are easy to differentiate because of the male pedipalps (Ubick et al. 2005). None of their ages were known, but experiments were conducted during the same time of year, from August to October. Transport back to the laboratory was done in plastic coolers (ca. 8,000 cc, l × w × h) containing moist leaf litter, and *H. cumberlandicus* and *M. ovalis* were used for experiments within 24 h after collection. All handling of specimens was done with an aspirator and forceps. Conditions for the laboratory experiments were 15° C ± 1° C, 97% RH (saturated K_2_SO_4_ solution; Winston and Bates 1960), in total darkness (0:24 L:D), maintained in an environmental room. Conditions at the study site in Laurel Cave, where experiments were conducted, were 12 ± 2° C and 98 ± 3% RH (thermo/hygrometer, Fisher Scientific, www.fishersci.com) and were consistent with measurements recorded in this region of the cave as reported by Hill (2003). Observations were made in red light.

### Uric acid, natural pheromone collection and target preparation

Uric acid was purchased commercially (Sigma Chemical Co., www.sigmaaldrich.com) and was diluted with HPLC grade acetone (Sigma); acetone also served as a control. All preparation and application of solutions were done using glass (miscellaneous glassware and 50 µ l pipettes, accuracy ± 0.25%, precision < 0.6%; Fisher). Filter paper was No. 3 Whatman (www.whatman.com) with an internal diameter of 9 cm. These filter paper discs served as targets in the attraction bioassays. All materials were handled using gloves and forceps to avoid contact with human skin oils. Any labeling on the filter paper disc was done with a graphite pencil (No. 2, Pentel, www.pentel.com).

Natural aggregation pheromone was collected by placing *H. cumberlandicus*, 10 at a time, in a plastic cooler (ca. 2,000 cc, l × w × h) containing a filter paper disc for 48 h, modified from aggregation pheromone collection by Nagel and Cade (1983) and McFarlane et al. (1983). Collection of natural pheromone was made from female adults as well as nymphs (mixed stages) and a total of 10 filter paper discs were prepared each from a separate population of *H. cumberlandicus* (10 from adults and 10 from nymphs).

To prepare the filter paper discs of uric acid, each dilution, [0.1M], [0.01M] and [0.001M] uric acid, was tested separately and applied (four applications of 50 µ l each, with air drying in between applications) at the center of the 9 cm filter paper disc and then allowed to air dry, making a spot application that did not exceed 3 cm in diameter. Three different sets of uric acid dilutions were prepared, and a fresh filter paper disc was used for each experiment. Control filter paper discs were those treated with 200 µl acetone, as well as untreated filter paper discs. Only one dilution of uric acid was tested at a time.

### Description of the bioassay

Experiments focused on a two-choice bioassay set up, providing a choice between two control filter paper discs (untreated or acetone) and two test filter paper discs (natural pheromone or dilution of uric acid) in a statistically valid four-quadrant block design (enlarged version of design from Arlian and Vyszenski-Moher (1995). The study area measured 1 m × 1 m and was divided into four equal sized quadrants (50 cm on each side of the midpoint); this was constructed using cotton thread (Anecot T-30, American and Efird, www.amefird.com) on the walls of the cave (securing the ends of the thread with poster putty) and on the outside of a clear plexiglass chamber (described below) in the laboratory tests. Each 50 cm × 50 cm quadrant was subdivided into four quadrants and the filter paper disc was placed at the center of the point of intersection (25 cm from the sides of each quadrant). The filter paper discs were placed in the study arena such that they alternated between quadrants: control (quadrant 1), test (quadrant 2), control (quadrant 3), test (quadrant 4). Therefore, a test quadrant was in between two control quadrants.

*H. cumberlandicus* were placed, ten at time, at the center of the arena. In the laboratory, the experiments were conducted in a clear plexiglass chamber (491,400 cc; 140 cm 1 × 130 cm w × 27 cm h) with the filter paper discs secured on the floor of the chamber that was then inverted so that the crickets were in an upside down position like they are in the cave; discs were at least 15 cm away from the walls of the chamber. Counts of *H. cumberlandicus* in the various quadrants (two control, two test/ study area) were made at 1 h and again at 6 h in both cave and laboratory settings. Filter paper discs were secured using nontoxic, unscented poster putty (Duck, Dial Co., St. Louis, MO).

### Sample size and data analysis

Each experiment represents observations on a total of 40 *H. cumberlandicus* each; thus 10 *H. cumberlandicus* per replicate, *n* = 4. In the experiments where *M. ovalis* was used, total sample size was 15 for each observation; thus, 3 *M. ovalis* per replicate, *n* = 5. Data were expressed as percentage of *H. cumberlandicus* or *M. ovalis* that were counted in the test quadrants. Untreated filter paper discs were used for comparison and to rule out potential directional left/right bias. Percentage data were arcsin transformed and analyzed using chi-square (χ^2^) statistics using 50% (20 *H. cumberlandicus*) or 47% (7 *M. ovalis*) as the expected (E) value, a = 0.05, d.f. = 1 (Sokal and Rohlf 1995).

## Results

### *H. cumberlandicus* responses

Within 1 h, 80% of female adult *H. cumberlandicus* in the laboratory bioassay crawled to filter paper discs that had been previously exposed and defecated upon by adult *H. cumberlandicus*, with the majority of them, 73%, remaining around these discs up to 6 h, all exhibiting significant attraction (Table 1). Nymphs reacted similarly at 1 h and 6 h with 70%) (28/40 *H. cumberlandicus*) and 78%) (31/40 *H. cumberlandicus*), respectively (c^2^ = 1.17; p > 0.05). Similar results were observed if adults were introduced to filter paper discs that had been exposed to nymphs and nymphs responded similarly to discs that had been exposed to adults, yielding high percentage attraction values ranging from 73% (29/40) - 83% (33/40). In all cases, *H. cumberlandicus* crawled directly to odor sources and stayed there. There was little movement between quadrants during the 6 h test period; in most cases, *H. cumberlandicus* made direct contact with the filter paper disc, with other individuals clustering around. The results to discs treated with uric acid produced significant attraction at [0.1M] uric acid at 1 h, but this was not significant at 6 h; and there was a significant response after 6 h at [0.001M] uric acid that was not significant at 1 h. Response to [0.01M] uric acid did not produce any significant stimulatory effect (Table 1). No dose reaction to increasing uric acid concentration was evident, and the response to uric acid was lower than to natural aggregation pheromone that was collected from *H. cumberlandicus* (Table 1). Over the range of uric acid concentrations, the combined effects of uric acid concentration compared favorably to controls (acetone-only and untreated filter paper) when averaged at 1 h (52% average of [0.1M], [0.01M] and [0.001M] uric acid responses) and 6 h (56% average of [0.1M], [0.01M] and [0.001M] uric acid responses) observations.

Similar results were obtained in the cave, with 68%) and 88% of *H. cumberlandicus* being attracted to filter paper discs that had been previously defecated upon and variable responses (low, if any, level attraction at [0.1M] and [0.01M] uric acid and even repellency at [0.001M] uric acid) to filter paper discs treated with uric acid. Filter paper discs that had been exposed to female adults were detected by nymphs, and filter paper discs that had been exposed to nymphs were attractive to female adults, with 78% (31/40) and 73% (29/40) attraction and arrestment, respectively, after 6 h (χ^2^ = 0.94; p > 0.05). There seemed to be stronger recruitment at 6 h than at 1 h in the cave with the natural *H. cumberlandicus* aggregation pheromone (Table 1). In both laboratory and cave settings, uric acid treatment failed to cause a retention effect, because there appeared to be more *H. cumberlandicus* movement to and from discs. Percentage attraction values for *H. cumberlandicus* in laboratory bioassays to acetone-only controls were 43% after 1 h and 55% after 6 h and were not significantly different from response to untreated filter paper, 48% and 40% attraction, respectively, (c^2^ = 1.31; p > 0.05; Table 1) with fairly even distribution, implying that there was little left/right bias in the experiment. Similar results to these controls in the laboratory bioassays were obtained in the cave (χ^2^ = 1.67; *p* > 0.05; Table 1), and these experimental values approximate the 50% expected (E) value that was used in the chisquare calculation. In conclusion, *H. cumberlandicus* respond by attraction and arrestment to filter paper discs that had been exposed to *H. cumberlandicus* but not to filter paper discs treated with uric acid.

**Table 1.**
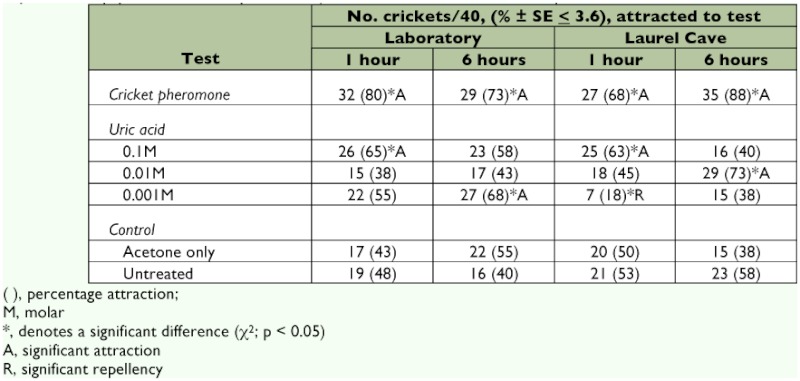
Cave cricket responses (female adult *Hadenoecus cumberlandicus*) to natural aggregation pheromone (cricket-exposed filter paper discs, cricket pheromone) and to uric acid as the main component of insect excreta.

**Table 2.**
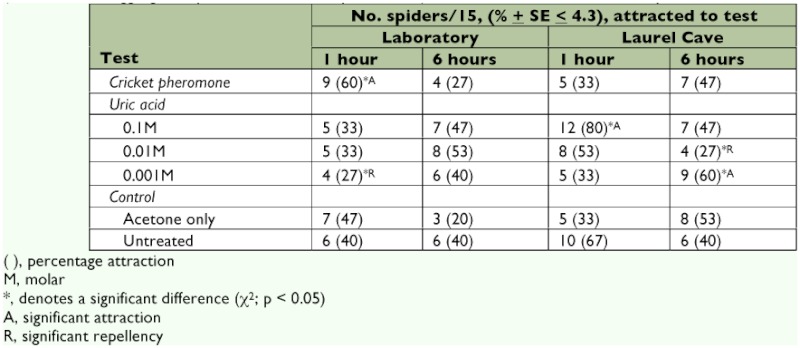
Spider responses (female adult *Meta ovalis*) to cave cricket (Hadenoecus *cumberlandicus*)-exposed filter paper discs (natural cricket aggregation pheromone, Cricket pheromone) and to uric acid as the main component of insect excreta.

### Spider responses

In laboratory bioassays, filter paper discs that had been defecated upon by female adult *H. ventured into the test quadrant. There was no dose effect to increasing uric acid concentration, and the number of *M. ovalis* in control quadrants (untreated and acetoneonly) was similar to values for *M. ovalis* that were counted in quadrants containing test attractants (Table 2). Percentage attraction to uric acid by *M. ovalis* across all concentrations ([0.1M], [0.01M], [0.001M] uric acid) averaged 31% at 1 h and 47% at 6 h, and this was similar to control results*.

In the cave, female adult *H. cumberlandicus-*contacted filter paper discs elicited no significant attraction activity to *M. ovalis* at 1 h or at 6 h (Table 2). At no time did *M. ovalis* change their course of direction in response to the test materials after they had been released in the bioassay arena. Recruitment to discs that had been exposed to nymphal *H. cumberlandicus* was similar to that toward filter paper discs made by exposure to adult *H. cumberlandicus* by not prompting significant activity by *M. ovalis* (data not presented). Uric acid-treated discs produced significant attraction by *M. ovalis* at [0.1M] uric acid at 1 h and at [0.001M] uric acid at 6 h, and repellency at [0.001M] uric acid at 6 h (Table 2). Results with increasing uric acid showed that it was not dose-dependent. When averaged across uric acid concentrations ([0.1M], [0.01M], [0.001M] uric acid), 55% of *M. ovalis* were in uric-acid treated areas at 1 h and 45% were present in uric acid-treated areas at 6 h, and these values were highly reminiscent to acetone-only and untreated controls (Table 2). In both laboratory and cave settings, the reaction by *M.ovalis* to controls were variable, ranging from 20% to 67%, and this reflects the movement and rather constant change in position in the bioassay arena by *M. ovalis* within the test arena which characterized the spider's reactions to the various *H. cumberlandicus*-associated test materials not occurring with any regular frequency. Collectively, counts of *M. ovalis* in treatment quadrants overlapped with counts of *M. ovalis* in control quadrants. The conclusion is that female adults of the cave orb weaver spider, *M. ovalis*, displayed no preference for filter paper discs that had been exposed to *H. cumberlandicus* or to uric acid.

## Discussion

In the cave cricket, *H. cumberlandicus*, the aggregation pheromone that operates is characterized by several key features, most of which are reminiscent of aggregation pheromone described in other orthopterans. The response involves attraction, indicating that there is long-range detection and thus a volatile active ingredient. There is a retention effect, where *H. cumberlandicus* exhibits reduced ambulatory activity (arrestment) on treated areas, leading to collections of numerous individuals, occasionally in direct contact with each other (little, if any, movement up to 6 h to adjacent quadrants after attraction). The response is not stage-specific, because nymphs react to excreta from female adults and female adults react to excreta from nymphs, which rules out classification of this chemical cue as a sex pheromone (furthermore, this test population of *H. cumberlandicus* is parthenogenic, where a sex pheromone has no purpose). Although speculative, the apparent derivation of aggregation pheromone from feces agrees with the abdominal origin of aggregation pheromone in the house cricket, *Acheta domesticus* (McFarlane et al. 1983) and the camel cricket, *Ceuthophilus secretus* (Nagel and Cade 1983). Although active components of *H. cumberlandicus* aggregation pheromone have not yet been identified (inconsistent results with uric acid rules it out experimentally), based upon related studies, it is conceivable that the active components are phenolic-based or organic acids derived from resident gut bacteria (McFarlane et al. 1983; Dillon et al. 2002).

Cave orb weaver spiders, *M. ovalis*, displayed no attraction responses, and at no time did they stop crawling and remain immobile when coming into contact with filter paper discs that previously had been exposed to *H. cumberlandicus*; i.e. this is earmarked by the number of *M. ovalis* in test quadrants that were similar to the number in control quadrants. Thus, there is no evidence that the aggregation pheromone of *H. cumberlandicus* contains an attractant or a retainer as a kairomone (host cue) to *M. ovalis*; therefore, *H. cumberlandicus* excreta (i.e. guano accumulation) apparently fails to be detected by *M. ovalis* as a signal of *H. cumberlandicus* prey quality (healthy, well fed, undiseased individuals), presence, or abundance. There is also no evidence that *H. cumberlandicus* aggregation pheromone contains any kind of repellent to *M. ovalis*, because no blatant avoidance behavior was displayed by *M. ovalis* when introduced to the *H. cumberlandicus* aggregation pheromone. Often when spiders are predators, the spider's excreta is detected and modifies the behavior of potential prey by stillness and reduced movements to go undetected, as though proceeding cautiously, described as a novel form of antipredator behavior (Persons and Rypstra 2001; Wilder and Rypstra 2004; Eiben and Persons 2007); prey (cricket) detects predators (spider) usually based upon the predator's diet (Kortet and Hedrick 2004). In fact, reduced movement in response to spider excreta is precisely how field crickets, *Gryllus integer*, react to the presence of the spider, *Hololena nedra* (Kortet and Hedrick 2004), and preliminary data testing spider excreta from *M. ovalis* suggest that this is also occurring in *H. cumberlandicus* (JB Benoit, The Ohio State University, Columbus, OH, unpublished data). In the present study, however, nymphs and female adults of *H. cumberlandicus* were shown to exhibit reduced activity (arrestment-retaining effect of aggregation pheromone) in response to their own excreta, thus it seems reasonable to suggest that the *H. cumberlandicus* aggregation pheromone similarly adds a useful defense function against predation by *M. ovalis* residing close to the *H. cumberlandicus* roost.

Cave crickets and cave spiders are characterized by losing water rapidly and drying out quickly (Studier et al. 1987; Hadley et al. 1981), and, as such, they have a high moisture requirement (hydrophilic water balance classification; Yoder et al. 2002). Indeed, localized sites selected within the cave where *H. cumberlandicus* aggregations are formed are where air is nearly water-saturated, temperature is stable, and air currents are reduced; thus, based upon a similar ecologic hydrophilic water balance profile, it is not surprising that *M. ovalis* shares the same habitat site in in that they have the same water loss problem. This study demonstrated that selection of these preferred sites by *H. cumberlandicus* is guided by an aggregation pheromone. This aggregation pheromone acts to recruit *H. cumberlandicus* to these sites and to retain them at these sites. As a product of cluster formation, the net transpiration (water loss) rates of individuals are greatly suppressed by nearly two-fold, leading to a group effect that facilitates water conservation (Yoder et al. 2002). Taken together, these considerations indicate that a few noteworthy attributes of *H. cumberlandicus* behavior are chemically-mediated by use of its aggregation pheromone: (1) habitat site selection within the cave, satisfying an absolute moisture requirement, presumably with access to outside food; (2) regulation of water balance via a resultant group effect; and, (3) perhaps defense against the predatory *M. ovalis* by antipredator behavior featuring reduced movement.
